# Music Preferences, Friendship, and Externalizing Behavior in Early Adolescence: A SIENA Examination of the Music Marker Theory Using the SNARE Study

**DOI:** 10.1007/s10964-017-0633-4

**Published:** 2017-01-18

**Authors:** Aart Franken, Loes Keijsers, Jan Kornelis Dijkstra, Tom ter Bogt

**Affiliations:** 1 Department of Developmental Psychology, Utrecht Centre of Child and Adolescent Studies, Utrecht University, Utrecht, the Netherlands; 2 Department of Developmental Psychology, Tilburg University, Tilburg, the Netherlands; 3 Department of Sociology, Interuniversity Center for Social Science Theory and Methodology, University of Groningen, Groningen, the Netherlands; 4 Department of Interdisciplinary Social Sciences, Utrecht Centre of Child and Adolescent Studies, Utrecht University, Utrecht, the Netherlands

**Keywords:** Music Marker Theory, Social network analysis, SIENA, Externalizing behavior, Early adolescence

## Abstract

Music Marker Theory posits that music is relevant for the structuring of peer groups and that rock, urban, or dance music preferences relate to externalizing behavior. The present study tested these hypotheses, by investigating the role of music preference similarity in friendship selection and the development of externalizing behavior, while taking the effects of friends’ externalizing behavior into account. Data were used from the first three waves of the SNARE (Social Network Analysis of Risk behavior in Early adolescence) study (*N* = 1144; 50% boys; *M*_age_ = 12.7; SD = 0.47), including students who entered the first-year of secondary school. Two hypotheses were tested. First, adolescents were expected to select friends based both on a similarity in externalizing behavior and music genre preference. Second, a preference for rock, urban, or dance, music types was expected to predict the development of externalizing behavior, even when taking friends’ influence on externalizing behavior into account. Stochastic Actor-Based Modeling indicated that adolescents select their friends based on both externalizing behavior and highbrow music preference. Moreover, both friends’ externalizing behavior and a preference for dance music predicted the development of externalizing behavior. Intervention programs might focus on adolescents with dance music preferences.

## Introduction

Music is a highly significant and meaningful medium, particularly in adolescence. Compared to older people, adolescents and young adults attribute more importance to music, and listen to music more often and in a wider variety of contexts (Bonneville-Roussy et al. 2013). Particularly in adolescence, music is not only important for mood management, but also for identity and social identity development (North and Hargreaves 1999; North et al. 2000). Music, its lyrics and visuals on TV, and the internet can be defining elements in the development of adolescent identity and social identity, particularly among those adolescents that are highly involved in music (North et al. 2000; Ter Bogt et al. 2010). Empirical evidence confirms that music is a factor in the formation of friendships, peer groups and peer culture (Selfhout et al. 2009; Steglich et al. 2006). Not only has music preference been linked to selecting friends with a similar music taste, particular preferences for several types of music have also been linked to the development of externalizing behavior. However, the impact of friends’ externalizing behavior on the link between music preference and externalizing behavior remains understudied.

Several types of music preference have been linked to externalizing behavior. For example, a preference for rock music, such as heavy metal, has been linked to externalizing behavior (e.g., Arnett 1996, Mulder et al. 2007; North and Hargreaves 2007; Selfhout et al. 2008; Ter Bogt et al. 2012; Tanner et al. 2008, Weinstein 1991). Similarly, a preference for urban music, such as rap or hip-hop, has been associated with externalizing behavior (Miranda and Claes 2004; Mulder et al. 2007; North and Hargreaves 2007; Selfhout et al. 2008; Tanner et al. 2008; Ter Bogt et al. 2012). Moreover, in ten countries across Europe a preference for dance music emerged as the most potent musical indicator for externalizing behaviors such as substance use (Ter Bogt et al. 2012). This latter study also indicates that currently, dance music was most consistently associated with several types of externalizing behavior (Ter Bogt et al. 2012).

In their Music Marker Theory, Ter Bogt et al. (2013) conceptualize the mechanisms through which music preferences translate into externalizing behavior. A fundamental hypothesis within Music Marker Theory states that it is not primarily the music itself or its lyrics that promote adolescent externalizing behavior. Instead, music preferences may work as a badge (Frith 1981), communicating values, attitudes and opinions. Adolescents are sensitive to the images that they themselves and their peers project and hold normative expectations about the characteristics of fans of particular musical styles (North and Hargreaves 1999; Rentfrow and Gosling 2006). Through showing their badge, adolescents identify themselves as belonging to or desiring to belong to specific peer groups and they may be drawn to other youth with similar taste. As such, peer involvement is thought to mediate between music and externalizing behavior. Through music, adolescents are drawn to specific crowds varying in externalizing behavior, which may influence their behaviors positively or negatively. In particular, listening to music types such as rock, urban, or dance music, is expected to lead to befriending others with similar music tastes. Among such friends, in turn, externalizing behavior is expected to occur more frequently and escalate more quickly (see Ter Bogt et al. 2013).

In support of the Music Marker Theory, several studies suggest that music preference plays a central role in friendship formation. From the seventies onward, a series of ethnographic studies among youth involved in sub-cultures revealed the central role of music in the structuring of peer groups or scenes (e.g., Willis 1978; Hebdige 1979; Bennett 2000, 2004). Indeed, similarity in music preferences has been shown to increase the likelihood of friendship (Frith 1981, Steglich et al. 2006). Selfhout and colleagues (2009) have shown that, over time, among stable friends there is high similarity in liking rock (divergent types of rock music), urban (i.e., Hip hop, R&B and reggae), pop/dance (mainstream music and the most popular forms of electronic dance music) and highbrow music (classical music and jazz). Furthermore, the study indicated that future friendships were created based on similarity in music preference. Thus, preferences for specific genres seem to indicate friendship similarity selection in early adolescence.

However, a proper test of the Music Marker Theory should control for effects of friends’ externalizing behavior. Indeed, like music preference, externalizing behavior has been shown to be important for both friendship creation and further development of externalizing behavior (see Moffitt 1993; Moffitt and Caspi 2001; Veenstra et al. 2013). Moreover, externalizing behavior itself might work as a badge, portraying a mature status among peers (Moffitt 1993). Therefore, it is important to take the effects of friends’ externalizing behavior into account when studying how music preference impacts friendship selection and the development of externalizing behavior.

To study the co-development of friendship and (externalizing) behavior, Stochastic Actor-Based Modeling (SABM) has been developed. Such modeling allows to simultaneously study both friendship similarity selection and influence processes (for an overview see Veenstra et al. 2013). Similarity selection takes place when adolescents select their friends based on similarities in behavior. Friendship influence processes take place when adolescents become more similar to their friends over time. It is important to disentangle these processes as they both lead to the same outcome: friends are similar to one another. Moreover, SABM controls for the increased likelihood of adolescents to reciprocate friendship, to become friends with classmates, or to become friends with their friends’ friends.

To our knowledge, only one study has simultaneously investigated the role of music preference and externalizing behavior in friendship formation and the development of externalizing behavior. Illustrating their Stochastic Actor-Based Modeling approach, Steglich et al. (2006) studied 129 adolescents and showed that, while taking friendship selection based on alcohol use into consideration, adolescents select their friends based on a similarity in classical music preference but not based on similarity in techno or rock music preference. Controlling for friendship selection based on similarity in music preference and the positive effects of adolescents’ friends’ alcohol use, the researchers did not find any effects for techno, rock, or classical music on the development of alcohol consumption.

### The Current Study

This study will investigate assumptions of the Music Marker Theory (Ter Bogt et al. 2013) that adolescents are likely to select friends based on similarity in music preference and that especially rock, urban, and dance music preferences are predictive of development of externalizing behavior. Listening to music types such as rock, urban, or dance music is expected to lead to befriending others with the same music preference and externalizing behavior is expected to occur more frequently among such friends, and as such, music preference may work as a badge, communicating values, attitudes and opinions (Frith 1981). Therefore, it was expected that adolescents who prefer rock, urban, or dance music are more likely to develop externalizing behavior. Most importantly however, since externalizing behavior is known to affect friendship selection and friends’ externalizing behavior is known to affect the development of externalizing behavior (e.g., Veenstra et al. 2013), this will be controlled for. Two hypotheses were tested. First, adolescents were expected to select friends based both on a similarity in externalizing behavior and music genre preference. Second, a preference for rock, urban, or dance, music types was expected to predict the development of externalizing behavior, even when taking friends’ influence effects on externalizing behavior into account.

## Methods

### Participants and Procedure

As this study focused on the entrance to secondary education, participants were 1144 first grade students (50% boys), aged between 11.1 and 15.6 at Time 1 (*Mean* 12.7, SD = 0.47). A total of 97% of participants were born in the Netherlands (as were 87% of their fathers and 88% of their mothers). Data stem from the SNARE (Social Network Analysis of Risk behavior in Early adolescence) study; a longitudinal project on the social development of early adolescents with a specific focus on adolescents’ involvement in risk behavior. Two secondary schools were asked and were willing to participate: one in the middle and one in the north of the Netherlands. Subsequently, all first- and second-year secondary school students (i.e., similar to 7th–8th grades in the US) from these schools were approached for enrollment in SNARE (2011–2012). All eligible students received an information letter for themselves and their parents, inviting them to participate in the study. If students wished to refrain from participation, or if their parents disagreed with their children’s participation, they were requested to send a note or email within 10 days to this effect. One year later (2012–2013), all new first year students were again approached for participation in the study. In total, 1826 students were approached for this study, of which 40 students (2.2%) refused to participate for several reasons, for example, the parent and/or adolescent had no interest, the adolescent was dyslectic, or it was too time consuming. A total of 1786 students participated in SNARE (*M* age Time 1 = 12.91 years, SD = 0.70, 50.1% male, 83.9% Dutch). Thus there were four samples, two cohorts coming from two schools (see also Dijkstra et al. 2015; Franken et al. 2015).

In September 2011, just when participants entered the first or second year of secondary school, we started with a pre-assessment. Subsequently, in 2012, all new first-year students also completed a pre-assessment. After the pre-assessment there were follow-up regular measurement waves in October (Time 1), December (Time 2), and April (Time 3). After 2 years (2011–2013), data collection was continued for another 2 years among the participating students.

During the assessments, a teacher and research assistants were present. The research assistant gave a brief introduction followed by the students filling in a questionnaire on the computer during class. The questionnaire contained both self-reports as well as peer nominations (participants could indicate who their friends were). Data were collected via the questionnaires using CS socio software (www.sociometric-study.com). This software was particularly developed for this study and allowed students to fill in sociometric questions. The assessment of the questionnaires took place during regular school hours within approximately 45 min. The students that were absent were, if possible, assessed within a month. The anonymity and privacy of the students were warranted. The study was approved by the Internal Review Board of one of the participating universities.

### Measures

#### Self-reported externalizing behaviors (Time 1–Time 3)

At all three time points, participants reported their engagement in three forms of externalizing behavior: Antisocial behavior, alcohol use, and tobacco use. Participants were asked if they engaged in the behavior during the previous month. Antisocial behavior was measured with 17 items by asking participants how often (between 0–12 or more times) they had been involved in 17 types of delinquent behavior; including stealing, vandalism, burglary, violence, weapon carrying, threatening to use a weapon, truancy, contact with the police, and fare evasion in public transport (see also, Nijhof et al. 2010; Van der Laan et al. 2010). For alcohol use, participants used a 13 point scale (ranging from 0 to over 40 times) to report on how many occasions they had consumed alcohol (Wallace et al. 2002). For tobacco use, participants used a seven-point scale (ranging from never to more than 20) to indicate how many cigarettes they had smoked daily (e.g., Monshouwer et al. 2011). Based on recommendations of Farrington and Loeber (2000), all three externalizing behavior scales were recoded as binary, indicating no engagement at all (0) or any engagement (1) in antisocial behavior, alcohol use, or tobacco use, respectively. As externalizing behaviors are known to cluster together during early adolescence (e.g., Monshouwer et al. 2012), an exploratory factor analysis (using maximum likelihood estimations and oblique rotation) revealed that the externalizing behaviors loaded on a single factor, explaining 55.3% of the variance. Therefore, a composite variable, representing the number of different externalizing behaviors participants engaged in (i.e., antisocial behavior, alcohol, tobacco use), was computed resulting in scores between zero (no externalizing behaviors) and three (all externalizing behaviors).

#### Friendship nominations (Time 1–Time 3)

Participants were asked to name their best friends. Participants could nominate friends in their own class and, in addition, friends from their grade. Grade networks were used for the current analyses. Thus, there were four networks (i.e., two schools and two cohorts per school).

#### Music preference (Time 1)

Participants were asked to indicate their music preferences. Fifteen music types were presented: international popular, Dutch popular, rock, alternative rock, heavy metal, gothic, rap/hip-hop, RnB, reggae, house/dance/trance, techno/dubstep, hardhouse, classical music, jazz, and folk. For each type of music, participants could tick a box, indicating their preference for this type of music. Thus for each music type participants had a score of either zero, indicating no preference, or one, indicating a preference for this type of music. A factor analysis (principle component analyses with oblimin rotation) revealed that 12 of these items can be meaningfully integrated into a five factor structure (see Table 1) similar to ones that that have been found in earlier studies on the structure of music preferences (e.g., Mulder et al. 2007; Rentfrow et al. 2011; Ter Bogt et al. 2012). On the basis of these results we created five overall music preference scores: popular, rock, urban, dance and highbrow music. Three less popular or less well known music types were not included in the current analyses: folk, reggae, and Dutch popular music.

**Table 1:** Exploratory factor analysis of music preferences at Time 1, with oblimin rotation

Music item	% Preference	Factor				
		Rock	Dance	Highbrow	Popular	Urban
Rock	23.5	0.78				
Heavy metal	8.2	0.69				
Alternative rock	8.1	0.68				
Gothic	2.5	0.48				
Techno/dubstep	16.7		0.68			
Hard house	16.4		0.66			
House/dance/trance	28.4		0.61			
Classical music	4.9			0.87		
Jazz	12.3			0.49		
International popular music	35.8				0.94	
Rap/hip-hop	41.2					0.82
RnB	15.0					0.70

*Note*. Loadings greater than (−) 0.32 are displayed. Kaiser-Meyer-Olkin Measure of Sampling Adequacy = 0.75. Bartlett’s Test of Sphericity: Chi^2^ 1296.22 (66), *p* < 0.01

### Analytic Strategy

Correlations between the music preference types and externalizing behavior were calculated. For each of the four friendship networks (i.e., 2 cohorts in 2 schools), descriptive statistics were also calculated including the average age, percentage of boys, average externalizing behavior level, and the percentage of absent participants of the networks. Furthermore, the Jaccard index, showing the relative stability of the friendship network over time, was calculated.

Participants were allowed to indicate who their friends were at each assessment and these nominations were combined into the same grade friendship networks within the school. The friendship network analyses were conducted using SIENA (Simulation Investigation for Empirical Network Analyses), version 4, in R. SIENA is an actor based model for the longitudinal co-evolution of social networks and individual behavior (Ripley et al. 2014). SIENA estimates changes in friendship nominations and externalizing behavior between two points in time; in this study, changes were calculated between Time 1 and Time 2 (Period 1), and between Time 2 and Time 3 (Period 2). While controlling for structural network effects (which take the structure of friendships in the network into account, such as the likelihood of being friends with the friends of your friends), SIENA estimates both network dynamics (i.e., changes in the network) and behavior dynamics (i.e., changes in behavior) longitudinally. The outcomes of SIENA analyses are based on an iterative Markov Chain Monte Carlo approach (Ripley et al. 2014; Snijders et al. 2010). For all analyses, the dependent variables consist of the network ties (friendship nominations) and the number of externalizing behaviors participants engaged in (antisocial behavior, alcohol use, and tobacco use). Two analyses were run. The first analysis only contained the estimated effects of music preferences on similarity selection (indicating whether participants were more likely to befriend each other based on similar music preferences) and the development of externalizing behaviors (indicating whether some music preferences are associated with a faster development of externalizing behavior). In the second analysis, externalizing behavior similarity selection effects (i.e., if participants select their friends based on similarity in externalizing behavior) and influence effects (i.e., if participants adapt their externalizing behavior based on their friends’ externalizing behavior) were added.

Both models contained commonly used structural network effects, effects which capture friendship relations (Ripley et al. 2014; Veenstra et al. 2013). Controlling for structural network effects is important as they explain why some adolescents become friends; for example adolescents are more likely to befriend the friends of their friends. Furthermore, additional network effects were added to optimally capture the friendship structure in the current networks. These included density (i.e., the number of present vs. absent friendship ties in the network), reciprocity (i.e., the likelihood to befriend those who befriend you), the likelihood to befriend friends of friends (transitive triplets), hierarchy (three-cycles), the likelihood for participants who receive many friendship nominations to receive extra friendship nominations (indegree popularity, square root version), the likelihood for participants who receive many friendship nominations to send extra friendship nominations (indegree activity, square root version), and the likelihood for participants who send out many friendship nominations to send out extra friendship nominations (outdegree activity, square root version); for more details see Ripley et al. (2014). To improve model fit, density and indegree popularity were allowed to vary between assessment periods. Furthermore, transitive reciprocated triplets were modeled to estimate the likelihood for triads (a group of three friends) to reciprocate friendships.

Additionally, several factors potentially affecting the friendship selection in the social networks (i.e., network dynamic effects) were estimated as covariates (see Veenstra et al. 2013). These effects are important as they may further explain friendship selection. Specifically, the effects of same-gender friendship selection (i.e., girls nominate girls, boys nominate boys; girls were coded as 0, boys as 1) were estimated as well as the effects of proximity by using adolescents’ classroom and school locations as covariates (School 1 consisted of four locations). The effects of gender and music preference on sending (called an ego effect) and receiving (called an alter effect) friendship nominations was also controlled for. To assess the first hypothesis that adolescents select friends based on similarity in music preference and externalizing behavior, selecting similar friends was modeled based on the different music preferences for the first and second analyses, and on externalizing behavior for the second analysis.

Behavior dynamic effects modelled changes in externalizing behavior, and include the effects from music preference and friends’ externalizing behavior on the development of externalizing behavior (see Veenstra et al. 2013). These effects are important as they capture the development of externalizing behavior and the impact of friendship and music preference on this development. In both analyses, these dynamics include the rate of change, and whether externalizing behavior changes conform to linear or quadratic trends. Furthermore, the effects from music preference (effect from) on the development of externalizing behavior were included in both analyses. These effects from music preference assess the second hypothesis, that rock, urban, or dance music preference predicts the development of externalizing behavior. The second analysis included effects from friends’ externalizing behavior (influence average alters); assessing whether participants change their externalizing behavior to become more similar to their friends’ externalizing behavior.

In a final step, after having estimated all these effects for the four networks separately, the estimated effects were summarized using the SIENA likelihood based method for meta-analyses (for more information see Ripley et al. 2014). The means and variances were normal, which indicates trustworthy outcomes of such a meta-analysis.

## Results

### Descriptive Statistics

Over the whole sample, there were significant and positive correlations (*p’*s *<* 0*.*01) between preferences for dance (*r* = 0.24) and urban (*r* = 0.09) music, and externalizing behavior. The correlations for highbrow (*r* = −0.10) and popular (*r* = −0.09) music with externalizing behavior were negative and significant (*p’*s *<* 0*.*01). A preference for rock music (*r* = 0.03) was not correlated with engagement in externalizing behavior.

Table 2 lists descriptive statistics for each of the four networks examined in this study. Results at Time 1 suggested that all four networks did not differ in age, and that there were only some small differences in gender distribution, and externalizing behavior. Table 2 also includes network characteristics for each cohort. Per network and measurement moment, there were between 1 and 5% absent participants during the assessments. The Jaccard index indicates the relative stability of each friendship network over time. The Jaccard indices were between 0.44 and 0.48, well within the desired range for longitudinal social network analyses (Veenstra et al. 2013). This indicates that friendships are relatively stable, while some changes in friendships occur. Therefore, it is possible to study changes in both friendship connections (making and losing friends) and to study changes in behavior among stable friends.

**Table 2:** Descriptive statistics of friendship networks for school 1 (cohort 1 *N* = 432, cohort 2 *N* = 390) and school 2 (cohort 1 *N* = 186, cohort 2 *N* = 136), Time 1–Time 3

Variable	School 1				School 2			
	Cohort 1		Cohort 2		Cohort 1		Cohort 2	
	Mean (SD)		Mean (SD)		Mean (SD)		Mean (SD)	
*Age*								
Time 1	12.65	(0.43)	12.65	(0.43)	12.66	(0.48)	12.70	(0.68)
*% boys*								
Time 1*	0.50^a^	(0.50)	0.48^ab^	(0.50)	0.47^ab^	(0.50)	0.61^b^	(0.49)
*Externalizing behavior*								
Time 1*	0.36^a^	(0.69)	0.47^b^	(0.82)	0.29^a^	(0.60)	0.34^ab^	(0.56)
Time 2	0.39	(0.68)	0.42	(0.75)	0.31	(0.66)	0.41	(0.69)
Time 3	0.44	(0.78)	0.51	(0.81)	0.42	(0.71)	0.47	(0.76)
*Rock preference*								
Time 1*	0.16^a^	(1.07)	−0.06^b^	(0.93)	−0.07^b^	(1.06)	−0.27^b^	(0.77)
*Dance preference*								
Time 1*	−0.16^a^	(0.94)	0.06^b^	(1.02)	0.14^b^	(1.08)	0.16^b^	(0.95)
*Highbrow preference*								
Time 1	0.02	(1.07)	−0.08	(0.92)	0.15	(1.03)	−0.03	(0.91)
*Popular preference*								
Time 1	0.02	(1.02)	0.00	(1.00)	−0.04	(1.01)	0.00	(0.94)
*Urban preference*								
Time 1*	−0.07^a^	(0.98)	−0.07^a^	(0.96)	0.27^b^	(1.10)	0.06^ab^	(0.99)
*Missing fraction*								
Time 1	0.01		0.03		0.05		0.01	
Time 2	0.01		0.04		0.03		0.01	
Time 3	0.03		0.03		0.02		0.02	
*Jaccard index*								
Time 1–Time 2	0.46		0.47		0.44		0.45	
Time 2–Time 3	0.46		0.48		0.44		0.45	

*Note.* *One-way ANOVA between group differences at *p* *<* 0.05. Within each time point (i.e., row), *Mean* scores with different superscripts differ significantly from each other at *p* *<* 0.05; calculated with a post-hoc Tukey Honestly Significant Difference test

### SIENA Estimates of Friends’ Influence

The outcomes of the meta-analysis of SIENA analyses of four networks are shown in Table 3 for both analyses. First, the structural network effects model the friendship network structure, and optimize the goodness of fit of the networks. It is important to model these effects as they help explain creation and maintenance of friendship. These effects were similar for both analyses and will therefore be explained once. There was a negative density effect (^1A^), indicating that participants are likely to be selective in their friendship nominations. There was a positive reciprocity effect (^1B^), indicating that participants are likely to reciprocate friendship nominations. There was a positive transitive triplet effect (^1C^), which shows that participants are likely to be friends with the friends of their friends. Furthermore, triads were less likely to have reciprocated ties than dyads, which is an indication of hierarchy in the network, as shown by a negative transitive reciprocated triplet effect (^1D^). Moreover, there was a negative three-cycle effect (^1E^). In combination with the positive transitive triplet effect this indicated that there was hierarchy in the networks (within triads few participants receive many nominations, while many participants receive fewer nominations). Particularly in period 2, between Time 2 and Time 3, we found a negative indegree—popularity effect (^1F^). This indicated that those with many friends were less likely to increase their number of friends. The negative effects of indegree—activity (^1G^) indicated that those participants who received many friendship nominations were less likely to send out nominations themselves. The outdegree activity (^1H^) was positive, indicating that those with a higher outdegree were more likely to increase the number of friends they select.

**Table 3:** Estimates of meta-analysis on four investigating networks similarity selection and influence effects based on music preference and externalizing behavior, in friendship networks at Time 1, 2, and 3

		Music only		Music and externalizing behavior	
*Network dynamics*					
^1^Outdegree (density)^1A^	Period 1	−2.17**	(0.15)	−2.25**	(0.12)
	Period 2	0.09	(0.12)	0.11	(0.12)
Reciprocity^1B^		2.58**	(0.11)	2.57**	(0.12)
Transitive triplets^1C^		0.52**	(0.02)	0.51**	(0.02)
Transitive reciprocated triplets^1D^		−0.43**	(0.04)	−0.43**	(0.04)
3-cycles^1E^		−0.06*	(0.02)	−0.06*	(0.02)
Indegree—popularity (sqrt)^1F^	Period 1	0.05	(0.06)	0.05	(0.06)
	Period 2	−0.13*	(0.04)	−0.14*	(0.04)
Indegree—activity (sqrt)^1G^		−1.03**	(0.09)	−0.99**	(0.09)
Outdegree—activity (sqrt)^1H^		0.15*	(0.04)	0.16*	(0.04)
^2^Sex received^2A^		−0.05	(0.07)	−0.05	(0.07)
Sex sent^2B^		−0.11	(0.11)	−0.02	(0.03)
Sex similarity selection^2C^		0.69**	(0.06)	0.69**	(0.05)
Class similarity selection^2C^		0.75**	(0.06)	0.76**	(0.06)
Location similarity selection^2C^		0.39	(0.03)	0.38	(0.04)
Rock received^2A^		−0.04	(0.02)	−0.04	(0.02)
Rock sent^2B^		−0.03	(0.02)	−0.02	(0.02)
Rock similarity selection^2C^		−0.20	(0.11)	−0.23	(0.11)
Dance received^2A^		−0.02	(0.03)	−0.02	(0.03)
Dance sent^2B^		0.05	(0.04)	0.03	(0.03)
Dance similarity selection^2C^		0.25	(0.14)	0.25	(0.13)
Highbrow received^2A^		0.05	(0.02)	0.05	(0.02)
Highbrow sent^2B^		0.03	(0.05)	0.04	(0.05)
Highbrow similarity selection^2C^		0.48*	(0.15)	0.52*	(0.14)
Popular received^2A^		−0.01	(0.02)	0.00	(0.02)
Popular sent^2B^		0.00	(0.03)	0.01	(0.03)
Popular similarity selection^2C^		0.00	(0.05)	−0.02	(0.05)
Urban received^2A^		0.00	(0.02)	0.00	(0.02)
Urban sent^2B^		0.07*	(0.01)	0.07*	(0.01)
Urban similarity selection^2C^		0.34*	(0.11)	0.34	(0.11)
Externalizing behavior received ^2A^				0.12	(0.04)
Externalizing behavior sent^2B^				0.21*	(0.05)
Externalizing behavior similarity selection^2C^				0.68*	(0.18)
*Behavior dynamics*					
^3^Externalizing behavior change period 1^3A^		1.38**	(0.13)	1.43**	(0.12)
Externalizing behavior change period 2^3A^		1.51**	(0.14)	1.54**	(0.15)
Externalizing behavior change linear^3A^		−1.27**	(0.07)	−1.26**	(0.07)
Externalizing behavior change quadratic^3A^		0.32**	(0.04)	0.21*	(0.05)
Effect from r\k on externalizing behavior^3B^		0.02	(0.04)	0.03	(0.05)
Effect from dance on externalizing behavior^3B^		0.19*	(0.04)	0.16*	(0.05)
Effect from highbrow on externalizing behavior^3B^		−0.12	(0.05)	−0.13	(0.06)
Effect from popular on externalizing behavior^3B^		−0.11	(0.05)	−0.09	(0.05)
Effect from urban on externalizing behavior^3B^		0.11	(0.04)	0.13	(0.05)
Externalizing behavior influence average alter^3C^				1.00*	(0.18)

*Note*. ^1^Effects estimating the structure of the friendship network. ^2^Effects estimating friendship selection. ^2A^Alter effects estimate the number of received friendship ties for participants with this characteristics. ^2B^Ego effects estimate the number of sent out friendship ties for participants with this characteristic. ^2C^Similarity effects estimate if participants base friendship selection on similarity of this characteristic. ^3^Effects estimating the change of behavior. ^3A^Estimating the development of externalizing behavior, and if this has a linear or quadratic shape. ^3B^Estimating the effect of this characteristic on the development of externalizing behavior. ^3C^Estimating the effect friends’ average externalizing behavior on the development of externalizing behavior

*p *<* 0.05; **p *<* 0.01

Second, to examine the first hypotheses that adolescents select their friends both on music preference and on externalizing behavior, the similarity selection effects were estimated for both analyses (Table 3). These effects indicate how adolescents create and maintain friendship, based on several characteristics such as gender or physical proximity (i.e., being in the same classroom). It is important to take such selection effects into account, as they help explain why friends are similar to one another. Three types of effects are important for this part of the model. First, received (or alter) effects (^2A^); which model whether participants are nominated as friends more frequently based on certain characteristics. Second, sent (or ego) effects (^2B^); which model whether participants with certain characteristics are more likely to nominate friends. Third, similarity selection effects (^2C^); which model whether participants are likely to select friends based on similarity in certain characteristics. The main effects of the control variables were generally consistent with prior research. While controlling for the number of friends adolescents select (sent effects) and the number of times they are selected as friends (received effects), participants’ selection of friends was significantly associated with similarity in gender and class. Therefore, participants were more likely to befriend peers with the same gender, and those who were part of the same class in school.

Partial support was found for the first hypothesis that friendship selection is based on music preferences. These effects indicate whether participants were more likely to befriend others who are similar to them in music preference or externalizing behavior. In the first analysis, without taking effects of friends’ externalizing behavior into account, participants were likely to select their friends based on a similarity in both highbrow and urban music preference (positive highbrow and urban similarity selection). Therefore, adolescents who had a preference for highbrow or urban music were more likely to select friends who also had a preference for highbrow or urban music, respectively. The second analysis also took friendship selection based on externalizing behavior into account. Participants were likely to select friends based on a similarity in externalizing behavior. While controlling for this friendship selection based on externalizing behavior (positive externalizing behavior similarity effect), similarity selection based on urban music became non-significant (*p* = 0.06), but the effect of friendship selection based on similarity in preferences of highbrow music remained significant (positive highbrow similarity selection effect). There was no selection based on similarity in other types of music.

Third, to enable a test of the second hypothesis, the change in externalizing behavior was estimated (Table 3). Behavior dynamics (i.e., changes in behavior) model the change in externalizing behavior. The first effects (^3A^) estimate the change of participants’ externalizing behavior. There was a negative linear effect, and a positive quadratic effect for the development of externalizing behavior. The combination of a negative linear effect and a positive quadratic effect indicates that externalizing behavior has a tendency to escalate once it develops: participants were likely to either engage in no externalizing behavior, or to engage in multiple externalizing behaviors.

To test the second hypothesis that rock, urban, and dance music preference would be associated with an increased likelihood to develop externalizing behavior (^3B^), effects from music preference on the development of externalizing behavior were tested. In the first analysis, without taking effects of friends’ externalizing behavior into account, music preference in rock, highbrow, popular, or urban music did not affect the development of externalizing behavior (non-significant effects from these types of music preference on externalizing behavior). However, preference for dance music was positively associated with the development of externalizing behavior. Thus, participants who had a preference for dance music were more likely to increase their externalizing behavior. In the second analysis, taking effects of friends’ externalizing behavior into account, participants were likely to be influenced by their friends’ externalizing behavior in the development of externalizing behavior (positive externalizing behavior average alter (^3C^)), and a preference for dance music still predicted an increase in externalizing behavior. This indicates that participants were likely to adapt their engagement in externalizing behavior to become more similar to their friends, and that there is an additional likelihood for participants who listen to dance music to develop externalizing behavior.

In sum, in partial support of the first hypothesis, adolescents were likely to select friends based both on music preference and on externalizing behavior. However, the selection of music preference was limited to a similarity in highbrow music, when taking selection based on externalizing behavior into account. Furthermore, partially supporting the second hypotheses, above and beyond the effects of friends’ externalizing behavior, adolescents who listen to dance music were likely to develop externalizing behavior. No friendship influence effects were found for preferences for rock or urban music.

## Discussion

Externalizing behavior is expected to occur more frequently and escalate more quickly among adolescents who prefer rock, urban, or dance music. According to the Music Marker Theory (Ter Bogt et al. 2013), such music preferences might work as a badge, communicating values, attitudes, and opinions (Frith 1981). Peer involvement is expected to mediate between music preference and externalizing behavior. Although similarity in music preference has been associated with friendship (e.g., Selfhout et al. 2009; Steglich et al. 2006), a proper test of the Music Marker Theory should control for the effects of friends’ externalizing behavior. Moreover, effects of friendship selection (adolescents befriend similar others) and influence (adolescents become similar to their friends) have to be disentangled. Therefore, this study set out to investigate whether (1) adolescents select friends based on music preference and/or on a similarity in externalizing behavior, and whether (2) adolescents’ preference for rock, urban, or dance music adds to the development of externalizing behavior beyond the influence effects of friends’ externalizing behavior. The results were based on two analyses, one excluding and one including the effects of externalizing behavior on friendship selection and on the development of externalizing behavior. The results provide partial support for both hypotheses. Adolescents were likely to select friends based on similarity in music preference, both on a preference for urban and on preference for highbrow music in the model excluding effects of friendship selection based on externalizing behavior. However, in the model taking similarity selection based on externalizing behavior into account, friendships selection was only based on a similarity in highbrow music preference. Moreover, irrespective of friends’ externalizing behavior, dance music was indicative for a faster increase in externalizing behavior. This study provides some support for claims by the Music Marker Theory (Ter Bogt et al. 2013) that music preference for certain music styles is an important indicator for externalizing behavior development, but this was limited to dance music only. Both a preference for dance music and friends who engage in externalizing behavior may influence adolescents’ engagement in externalizing behavior.

Although it was expected that a preference for rock, urban, and dance music would all be associated with externalizing behavior development, only dance music significantly predicted the development of externalizing behavior. Interestingly, dance music has recently also been specifically identified as most consistently associated with several types of externalizing behavior in Europe (Ter Bogt et al. 2012). Therefore, our findings support the idea that dance music, rather than rock or urban music, is currently the music type most associated with externalizing behavior. Moreover, the finding that dance music predicts future externalizing behavior also adds to the claim that music preference works through a badge rather than directly through the music itself or the lyrics. Dance music’s lyrics and visuals on TV are much less associated with externalizing behavior, compared to for example, rock or urban music. Thus, the values, attitudes, and opinions transmitted through dance music might be important especially to adolescents.

This study took into account that adolescents are likely to befriend peers who are similar in music preference and in externalizing behavior and controlled for the effect of friends’ externalizing behavior on participants’ own externalizing behavior. Even while controlling for these alternative explanations of the development of externalizing behavior, music preference predicted future externalizing behavior. This is in line with the findings of Ter Bogt and colleagues (2013). However, this is in contrast to the study of Steglich et al. (2006) who did not find such influence effects from music preference while investigating alcohol use among 129 adolescents of the age of 13 year, using three yearly assessments. This may possibly be because Steglich et al. (2006) focused on alcohol use rather than a more global construct of externalizing behavior. Furthermore, there may have been too few participants, other music preferences such as hip-hop or RnB could have been more important at the time of the study (data was collected starting 1995), or measurement moments could have been too far apart. During secondary school, friends may change their classrooms from one year to another.

Friendship selection was not based on similarity in rock, dance, popular, or urban music preferences when taking friendship selection based on externalizing behavior into account. Urban music, however, was associated with friendship selection if friendship selection on externalizing behavior was not taken into account. Thus, friendship selection based on externalizing behavior partially explains friendship selection based on a preference for urban music. In both models with and without externalizing behavior, friendship similarity selection was based on externalizing behavior and on a preference for highbrow music. The selection effect based on highbrow music is in line with the finding of Steglich and colleagues (2006) and might indicate that there is a strong basis for early adolescents to select one another on a similarity in preference for highbrow music. When looking at these findings from the perspective of music and externalizing behavior working as badges (Frith 1981), it is possible that externalizing behavior takes over the role of badge that urban music would otherwise have. Possibly the badge of engagement in externalizing behavior, which Moffitt (1993) expects to signal social maturity, is more prominent than the musical badge in early adolescence. This would help explain why similarity selection based on a preference for urban music lost its significance when taking friendship selection based on externalizing behavior into account, as urban music preference was positively associated with externalizing behavior. Highbrow music was negatively associated with externalizing behavior, which may help explain why, next to externalizing behavior, adolescents select friends with a similar preference for highbrow music.

It would be interesting to further investigate these friendship similarity selection processes, and their underlying motivations. For example, comparing which roles group formation based on music preference and externalizing behavior fulfill would allow a better understanding of these underlying motivations. Both externalizing behavior and urban music might serve to signal friendship selection based on a more mature status or badge, but there might also be different reasons for such friendship selection. For example, in the case of highbrow music preference, music preference might help adolescents obtain a different social goal. Future studies could further investigate these mechanisms.

The main strength of this longitudinal network study is that both music preference and externalizing behavior were estimated while taking friendship, embeddedness of friendship in networks, and changes of friendship and externalizing behavior into account. This was done every 3 months after adolescents entered a new network of friends; thus the effects found in this study are likely based on current music preference and externalizing behavior rather than pre-existing friendships. Therefore, this provides a stringent test of the assumption that music preference plays an important role explaining both friendship selection and the development of externalizing behavior during adolescence. Moreover, these analyses were done in two models: one with and one without the effects of externalizing behavior. A second strength of the current study is that we identified profiles of music preference, using principal component analyses. This allowed adolescents to have a profile of music preference, which is more informative compared to basing music preference solely on some exemplary items.

As any study, this study also has some limitations. One important limitation is that changes in music preference were not accounted for. It would be interesting to investigate how externalizing behavior and friendship affect changes in music preferences, and how these changes in turn impact externalizing behavior and friendship. Secondly, although friendship similarity selection was modeled, the Music Marker Theory might even better explain effects based on friendship groups or cliques, rather than individual friendships. Thirdly, this study focused on the occurrence of adolescents’ externalizing behaviors rather than the frequency with which adolescents engage in such behaviors. Future studies should investigate this frequency, perhaps during later years in adolescence when there is more engagement in externalizing behavior. In such a sample of late adolescents, it might also be possible to compare friendship influence processes with regard to different kinds of externalizing behaviors. Moreover, the current study focused on music preference and the influence of friends. Future studies should take other important aspects for the development of externalizing behavior into account, such as self-control (see Gottfredson and Hirschi 1990) or pubertal development (Dijkstra et al. 2015). With the complexity of these findings, future studies should aim to study the impact of musical preference on externalizing behavior in alternative ways. Comparing more detailed differences in music preference, for example differentiating between different types of dance music, might build on these findings and be a good start to further study how music is associated with externalizing problems.

## Conclusions

This study showed that both music preference and friends’ externalizing behavior are important in explaining the spread of early adolescent externalizing behavior, and that they do so in an additive manner. Both dance music and friends’ externalizing behavior predicted increases in externalizing behavior. Therefore, it is important to take adolescent preference for dance music into account when studying the development of early adolescent externalizing behavior. Adolescents who listen to dance music especially, are more likely than their peers to develop externalizing behaviors. Prevention programs could aim prevention efforts at adolescents who listen to dance music, as they can be easily targeted through their music stations or dance related events.
